# Kras as a key oncogene and therapeutic target in pancreatic cancer

**DOI:** 10.3389/fphys.2013.00407

**Published:** 2014-01-21

**Authors:** Meredith A. Collins, Marina Pasca di Magliano

**Affiliations:** ^1^Program in Cellular and Molecular Biology, University of MichiganAnn Arbor, MI, USA; ^2^Department of Surgery, University of MichiganAnn Arbor, MI, USA; ^3^Department of Cell and Developmental Biology, University of MichiganAnn Arbor, MI, USA

**Keywords:** Kras, pancreatic cancer, PanIN, therapeutics, MAPK, PI3K/AKT/mTOR

## Abstract

Pancreatic cancer is one of the deadliest human malignancies and little progress has been achieved in its treatment over the past decades. Advances in our understanding of the biology of this disease provide new potential opportunities for treatment. Pancreatic cancer is preceded by precursor lesions, the most common of which are known as Pancreatic Intraepithelial Neoplasia (PanIN). PanIN lesions, which are the focus of this review, have a high incidence of Kras mutations, and Kras mutations are a hallmark of the late-stage disease. We now know from genetically engineered mouse models that oncogenic Kras is not only driving the formation of pancreatic cancer precursor lesions, but it is also required for their progression, and for the maintenance of invasive and metastatic disease. Thus, an enormous effort is being placed in generating Kras inhibitors for clinical use. Additionally, alternative approaches, including understanding the role of Kras effector pathways at different stages of the disease progression, are being devised to target Kras effector pathways therapeutically. In particular, efforts have focused on the MAPK pathway and the PI3K pathway, for which inhibitors are widely available. Finally, recent studies have highlighted the need for oncogenic Kras to establish feedback mechanisms that maintain its levels of activity; the latter might constitute alternative ways to target Kras in pancreatic cancer. Here, we will review recent basic research and discuss potential therapeutic applications.

## Kras in pancreatic cancer initiation and maintenance

The association of mutant Kras with pancreatic cancer was established decades ago (Almoguera et al., 1988; Smit et al., 1988); the most common mutation is one amino-acid substitution in position 12 of the Kras protein, leading to a glycine (G) to aspartic acid (D) substitution, although other variants, such as G to V are also common (for review see Pylayeva-Gupta et al., 2011). The mutations compromise the ability of the Ras protein to hydrolize GTP to GDP, thus effectively locking the protein in an active conformation. Recent tumor genome sequencing studies have established the prevalence of mutant Kras in Pancreatic Intraepithelial Neoplasia (PanINs), the most common precursor lesions (Kanda et al., 2012), and in pancreatic cancer (Jones et al., 2008; Biankin et al., 2012) with increased precision. Results from these studies confirm the notion that over 90% of early stage PanIN and invasive tumors express mutant Kras. It is interesting to note, however, that efforts to use mutant Kras (detected in the pancreatic juice) as biomarker of pancreatic cancer have not been successful, as Kras mutations are common in an age-dependent manner even in people who are devoid of pancreatic malignancy (Yakubovskaya et al., 1995; Lu et al., 2002; Yan et al., 2005; Parsons and Meng, 2009). Moreover, the likelihood of low-grade PanINs that progress to pancreatic cancer is as yet unclear, as those pre-neoplastic lesions also occur in the general population at a much higher rate than pancreatic cancer (Singh and Maitra, 2007).

## Initiation

Insight into the functional role of oncogenic Kras during the onset pancreatic cancer has been obtained using genetically engineered mouse models of the disease. Several different approaches were used to target expression of oncogenic Kras to the mouse pancreas, and discussing all of them goes beyond the scope of this review. Arguably, the first models to mimic the human disease, specifically the progression of PanINs to invasive cancer, have been based on the expression of oncogenic Kras in a tissue-specific manner, and from the endogenous Kras locus. The endogenous Kras-based models rely on Pdx1-Cre (Hingorani et al., 2003) or Ptf1a-Cre (Kawaguchi et al., 2002) to obtain tissue-specific expression of Kras by Cre-mediated recombination of a stop cassette placed in the Kras locus (Jackson et al., 2001). Both Cre strains drive expression of the recombinase across all the pancreatic lineages. However, Pdx-Cre is also expressed in the duodenum, while Ptf1a-Cre is exclusively pancreas-specific within the gastrointestinal tract. Pdx1-Cre;LSL-Kras^G12D^ and Ptf1a-Cre;LSL-Kras^G12D^ mice are generally referred to as KC (Hingorani et al., 2003; Olive and Tuveson, 2006). KC mice express oncogenic Kras from the earliest stages of pancreatic embryonic development. However, they have a normal pancreas at birth. PanINs are first noticed shortly after weaning, and they progress in grade and number over time. Thus, KC mice provided the first line of evidence that mutant Kras was necessary and sufficient for the initiation of pancreatic cancer. Progression to invasive pancreatic cancer occurs sporadically, and usually in older animals. KC mice have opened a whole field of pancreatic cancer research, as they have served as the basis to interrogate other signaling pathways and genetic events leading to pancreatic carcinogenesis (reviewed in Morris et al., 2010b), as well as the effect of environmental factors. The slow progression to invasive disease, however, limited the use of these mice for pre-clinical studies. Based on the observation that tumor suppressor genes are usually lost or inactivated in the human disease, KC mice have been crossed with loss-of-function or mutant allele for Ink4a (Aguirre et al., 2003) or p53 (Hingorani et al., 2005). The latter, commonly known as KPC mice, are currently the most promising preclinical model in pancreatic cancer, thanks to the development of imaging techniques (such as high resolution ultrasound) that allow individual animals to be evaluated for the presence and size of tumors (Olive et al., 2009). While their response to standard of care therapies for pancreatic cancer can resemble that observed in human patients (Singh et al., 2010), it should be acknowledged that this may not always be the case and preclinical studies should be translated to human patients with caution. For instance, the promising response observed in GEMs upon gemcitabine and Hedgehog pathway inhibitors in KPC mice (Olive et al., 2009) did not hold true in human patients enrolled in a recent clinical trial (http://phx.corporate-ir.net/phoenix.zhtml?c=121941&p=irol-newsArticle&ID=1653550&highlight=).

## Pancreatitis and oncogenic Kras

The observation that Kras mutations occur at much higher frequency than pancreatic cancer in humans is recapitulated in mouse studies, where—although every single pancreatic epithelial cell expresses mutant Kras from the early pancreas development—PanIN lesions occur sporadically and only several weeks after birth. Thus, it emerges that additional events, whether genetic or epigenetic, need to occur to initiate carcinogenesis. One of the best established risk factors for pancreatic cancer is pancreatitis. Chronic pancreatitis patients have an elevated risk of developing pancreatic cancer (Lowenfels et al., 1993; Malka et al., 2002; Whitcomb and Pogue-Geile, 2002). The potential effect of acute pancreatitis on carcinogenesis is not as well understood in humans. However, it is possible that even low, subclinical levels of local or systemic inflammation might promote the formation of PanINs, in presence of mutant Kras. In mice, both acute and chronic pancreatitis have been shown to synergize with oncogenic Kras to drive the onset of carcinogenesis. In mice that activate the expression of the Kras^G12V^ mutant in the adult pancreas, carcinogenesis only occurs upon induction of chronic (Guerra et al., 2007) or acute (Guerra et al., 2011) pancreatitis. The latter finding has been recapitulated in the recently described iKras^*^ mouse model of pancreatic cancer (Collins et al., 2012a), which will be described in more detail later in this review. Moreover, even in KC mice, the induction of acute pancreatitis leads to rapid and extensive PanIN formation (Carriere et al., 2009, 2011; Morris et al., 2010a). Taken together, the current literature suggests that genetic events and environmental changes cooperate to induce pancreatic carcinogenesis. However, how these elements contribute to cancer onset in human is not yet fully understood.

## Maintenance

Human pancreatic cancer cell lines have been extensively used to study the disease. They have also provided the first system to address the role of oncogenic Kras in tumor maintenance, and to gain insight in the biologic role of Kras signaling in tumors. Most cell line studies have relied on traditional cell culture systems, thus they did not recapitulate the three-dimensional relationship within the tumor nor the interactions between tumor cells and their microenvironment. Nevertheless, knock-down studies have identified Kras-dependent and independent human cell lines, and identified a Kras “signature.” Amplification of not only Kras, but also upregulation of genes involved in cell survival as well as epithelial differentiation are key characteristics found in the Kras-dependency signature and are predictive of Kras “addiction” (Singh et al., 2009). More recently, ductal and quasi-mesenchymal subsets of primary human tumors were identified (Collisson et al., 2011). In addition to different morphology and expression of ductal genes vs. mesenchymal-lineage genes—hence the nomenclature—the two subsets differed in their dependence on oncogenic Kras. In fact, ductal cells were Kras-dependent both *in vitro* and when transplanted into immune-compromised mice, while cell lines with quasi-mesenchymal characteristics were Kras-independent.

**Figure 1:**
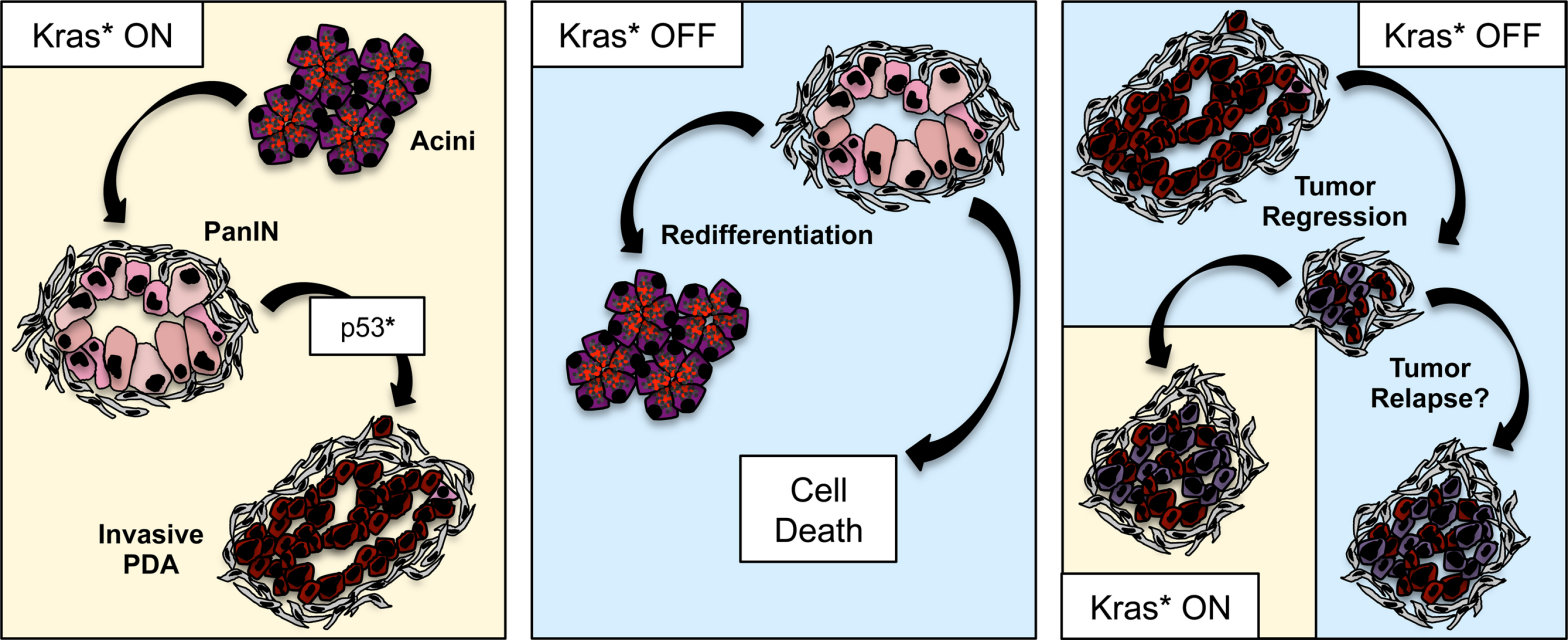
**Oncogenic Kras in pancreatic cancer progression and maintenance**. Oncogenic Kras drives PanIN formation and—in combination with loss or mutation of tumor suppressors such as p53—progression to invasive adenocarcinoma. Inactivation of oncogenic Kras at the PanIN stage leads to regression of the lesions, through a mechanism that includes cells death as well as re-differentiation of PanIN cells to acini. Inactivation of oncogenic Kras in metastatic tumor leads to tumor regression; however, a subset of tumor cells survive Kras inactivation, possibly entering a dormancy status, and setting the stage for tumor relapse.

Finally, the question of Kras dependency in pancreatic cancer has been addressed in genetically engineered mice. The iKras^G12D^ (iKras^*^) model, recently described (Collins et al., 2012a), allowed for the first time to express oncogenic Kras in an inducible, tissue-specific and reversible manner. Thus, oncogenic Kras could be turned off at different stages of carcinogenesis and the effects studied. Kras inactivation in PanINs resulted in rapid tissue remodeling: the PanIN cells re-differentiated into acinar cells, and the desmoplastic stroma was cleared through an as yet not fully understood mechanism. Kras inactivation in advanced PanINs led to massive epithelial cell death, together with some redifferentiation of acinar cells that then became proliferative and partially repopulated the pancreas parenchyma. A similar effect was seen with Kras inactivation in tumors. A further study including metastatic pancreatic cancer (Collins et al., 2012b) and *in vivo* imaging showed regression of primary tumors and metastases. However, a subset of the tumor cells survived in a dormant state, but could resume rapid growth upon Kras re-activation. In terms of translational potential of these studies, it is worth noting that Kras-independent tumors were not observed in this mouse model, potentially indicating a mouse vs. human difference. However, the tumors did broadly fall in a ductal and a quasi-mesenchymal category, both of which required Kras for growth *in vivo*. Primary tumor cell lines derived from iKras^*^ mice carrying a mutant allele of p53 were Kras-independent for their growth in two-dimensional cell culture, but required Kras for three-dimensional growth. Lastly, the persistence of some tumor cells upon Kras inactivation indicates that Kras inhibitors—were they to become available—might not completely “cure” pancreatic cancer. The concern is for the surviving cells to eventually either become resistant to Kras, or grow back when Kras inhibition is released. Thus, it will be important in the future to understand the mechanism(s) that allow a subset of tumor cells to survive Kras inhibition and achieve long-term dormancy (Figure 1).

## Biologic role of Kras in pancreatic cancer cells (metabolism, macropinocytosis, regulation of the stroma and the inflammatory response)

While the link between mutant Kras and pancreatic cancer has been long established, the biological function of Kras signaling in pancreatic cancer cells is still being investigated, and some important progress in this area has been achieved only very recently. iKras^*^ mice were used to perform microarray expression analysis experiments. Interestingly, several genes involved in metabolism were identified as regulated by Kras (Ying et al., 2012). In fact, Kras appears to induce the switch between a mostly aerobic metabolism, characteristic of the healthy pancreas, with an anaerobic mechanism mainly through the lactic acid pathway, which is associated with cancer cells. Additionally, it has also been shown that Kras regulates glutamine metabolism through non-canonical methods to aid in the maintenance of the tumor cell's redox state (Son et al., 2013). Moreover, the activation of the reactive oxygen species detoxification program was shown to be regulated by Kras (Denicola et al., 2011). Reactive oxygen species (ROS) are thought to be mutagenic and promote cancer, while the ROS detoxification program is thought to be beneficial to the cell by clearing away the toxic compounds; however, the data presented by DeNicola et al. contradict this concept. Specifically, the authors show that oncogenic Kras promotes tumorigenesis by inducing expression of NRF2, a key component in the ROS detoxification program, and that reducing ROS levels is necessary for PanIN/cancer progression (Denicola et al., 2011). Thus, if the ROS detoxification program is impaired as it occurs in mice lacking NRF2 expression, then pancreatic carcinogenesis is inhibited, indicating that this is a fundamental mechanism to allow cells to bypass early barriers to carcinogenesis. Kras also regulates other key cellular functions related with the elevated energy needs to cancer: macropinocytosis, induced by oncogenic Kras, allows the cancer cells to acquire albumin from the surrounding extracellular space, and use it to produce Krebs cycle intermediates (Commisso et al., 2013).

In addition to intracellular factors regulated by Kras, the interactions between the tumor cells and their microenvironment are also controlled by this oncogene, although the full extent of this regulation and the mechanisms underlying it are as yet poorly characterized. In iKras^*^ mice, inactivation of oncogenic Kras at any stage of carcinogenesis leads to loss of proliferation and Smooth muscle actin expression in the stroma (Collins et al., 2012a). Those changes are consistent with the conversion of an active stroma to scar-tissue like fibrosis. One of the signals mediating the interaction between the tumor cells and the surrounding fibroblasts within the stroma might be Sonic Hedgehog, one of the Hedgehog pathway ligands that is secreted by the tumor cells (Berman et al., 2003; Thayer et al., 2003), and activates signaling in a paracrine manner in fibroblasts (Yauch et al., 2008). However, it is likely that additional signals regulate the interactions between Kras-expressing epithelial cells and the surrounding microenvironment.

The formation of PanINs, in humans and mice, is accompanied by infiltration of immune cells. Interestingly, the subsets of immune cells that infiltrate are different than the immune cells normally present within the pancreas, and include abundant regulatory T cells and myeloid derived suppressor cells, while excluding cytotoxic T cells (Clark et al., 2007). Thus, Kras expressing epithelial cells establish early on an immune suppressive environment, that allows tumor growth. The functional role of the infiltrating cells is an area of active investigation, given the potential to use modulation of the immune response in cancer therapy (for review see Jaffee et al., 2001; Clark et al., 2009; Vonderheide and Bayne, 2013).

## The search for Kras inhibitors

The recent data highlighting the importance of Kras in the maintenance of pancreatic cancer demonstrates the necessity for the development of Kras inhibitors. While the ideal mechanism to prevent Kras signaling would be to directly block the GTP-binding site of Kras, an effective small molecule inhibitor has yet to be identified. Instead, multiple groups have investigated the efficacy of targeting Kras indirectly.

Following translation, Kras is farnesylated allowing the protein to associate with the membrane thus bringing it into contact with Ras activating proteins. At the membrane Kras is activated by Ras-GEFs, guanine nucleotide exchange factors, specifically SOS, which aids in Kras binding GTP. Farnesyltransferase inhibitors (FTIs) were initially thought to be the silver bullet for Kras inhibition due to the requirement for this post-translational modification for the subsequent activation of Kras. A number of FTIs have been tested in the clinic, such as Lonafarnib and Tipifarnib, and have predominantly proven unsuccessful for Kras-driven tumors (for review see Appels et al., 2005). This lack of success can be attributed to the differences between the three Ras proteins. The preclinical studies that induced most of the excitement for the potential of FTIs were performed on Hras-dependent tumors (Kohl et al., 1995). In contrast to Hras, Kras, and to some extent Nras, can be geranyl-geranylated upon inhibition of the farnesyltransferase (Whyte et al., 1997). This alternate post-translational modification provides Kras with an escape mechanism, enabling it to continue to associate with the membrane and its activating proteins.

This failure of the FTIs to successfully prevent Kras activity and subsequent downstream signaling has prompted exploration of other means of Kras inhibition. Recently, multiple groups have investigated strategies to prevent Kras from reaching the membrane. One such inhibitor, Deltarasin, is a small-molecule that binds to the farnesyl-binding pocket of PDEδ (Zimmermann et al., 2013). PDEδ interacts with farnesylated-Kras and aids in the translocation of Kras to the membrane (Chandra et al., 2012). Therefore, interaction between Deltarasin and PDEδ allows for the farnesylation of Kras but prevents Kras from reaching the membrane. Another inhibitor, Salirasib, targets the localization of Kras to the membrane. In contrast to PDEδ inhibition, Salirasib blocks Kras activity by dislodging the farnesylated protein from the membrane (Weisz et al., 1999). Importantly, Salirasib has already shown potential as a Kras inhibitor in preclinical and clinical trials of pancreatic cancer (Laheru et al., 2012).

**Figure 2:**
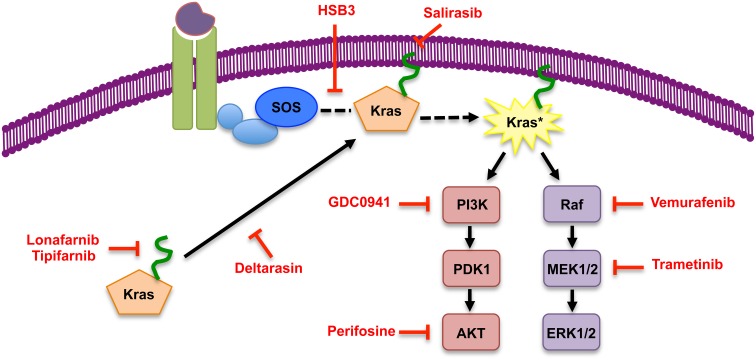
**Inhibitors of Kras and of its effector pathways**. Simplified scheme of Kras signaling, with representative inhibitors of signaling components indicated in red. The inhibitor list is not comprehensive.

In addition to the inhibitors designed to block Kras from reaching the membrane, others have devised means to prevent Kras activity at the membrane by inhibiting the interaction between Kras and its Ras-GEF SOS. Patgiri et al. have designed a small molecule alpha-helix, using the hydrogen bond surrogate (HBS) approach, that interferes with the Ras-SOS interaction and therefore blocks the exchange of GDP for GTP, subsequently decreasing Ras activity (Patgiri et al., 2011). Recently, it has been shown that Kras is acetylated, in addition to the aforementioned post-translational modifications, and the presence of the acetyl group alters SOS ability to exchange GDP for GTP. Future work will identify the role acetylation plays in the activity of mutant Kras and hence downstream cellular changes. This new discovery of Kras acetylation highlights another mechanism that can possibly be exploited to target and inhibit Kras activation (Figure 2).

This recent surge of inhibitors that prevent Kras activity indirectly is extremely exciting and promising. These inhibitors allow for the normal post-translational modification of the oncoprotein, removing need for alternative processing, but prevent its activity by interrupting its localization to the membrane or block the necessary interactions with its activating proteins. Clinical success of such inhibitors could dramatically change the therapeutic outlook for pancreatic cancer patients.

## Downstream effectors of Kras and the biology of pancreatic cancer

Several effector pathways are activated downstream of Kras, in a context and tissue-specific dependent manner (for review, Bar-Sagi, 1992; Campbell et al., 1998, 2006, 2007; Cox and Der, 2002; Pylayeva-Gupta et al., 2011). The two pathways that have been studied most in detail in pancreatic cancer are MAPK and PI3K signaling. The MAPK pathway consists of a kinase cascade, whereby Raf kinases are activated by Kras and, in turn, activate MEK1/2. MEK kinases phosphorylate and activate ERK1/2 (for review, see Dhillon et al., 2007). MAPK signaling is active in PanIN lesions as well as in late-stage pancreatic cancer, both in human tumors and in mouse (Hingorani et al., 2003). Two key lines of evidence indicate the importance of this pathways during carcinogenesis: forced activation of MAPK signaling through overexpression of a constitutive form of Raf leads to PanIN/PDA formation and, conversely, blocking MAPK signaling blocks the onset of carcinogenesis (Ardito et al., 2012; Collisson et al., 2012). In contrast, expression of a constitutively active form of PI3K did not induce PanIN formation (Collisson et al., 2012). However, the latter finding has been contested more recently, as in a different context activation of PI3K signaling does induce pancreatic carcinogenesis, and inhibition of this pathway blocks carcinogenesis (Eser et al., 2013). It is therefore possible that both pathways are important during disease formation. The question remains, however, as to the relative importance of those signaling pathways both during cancer formation and in advanced tumors. Moreover, eventual feedback mechanisms linking them have not been explored in pancreatic cancer, but they have been identified in other tumors and could lead to acquired resistance to inhibitors (Emery et al., 2009; Corcoran et al., 2010; Dai et al., 2011).

Given that both MAPK and PI3K signaling are active in a large number of tumor types, small-molecule inhibitors for each pathway have been developed. The MAPK pathway can be blocked at the level of Raf (such as Vemurafenib, PLX4032); however, recent studies have highlighted the efficacy of Raf inhibitors is highly dependent on the cellular context. Raf inhibition is effective in Raf mutant tumors (such as melanoma); in contrast, the use of Raf inhibitors in Kras mutant tumors results in the paradoxical upregulation of MAPK signaling. Specifically, in tumors bearing wild-type Raf but mutant Ras (such as pancreatic cancer), Raf inhibitors create feedback activation of MAPK signaling by inducing dimerization of cRaf with BRaf and interaction with the oncoprotein Kras-GTP (Hatzivassiliou et al., 2010; Heidorn et al., 2010; Poulikakos et al., 2011). Therefore, MEK inhibition has emerged as a more promising therapeutic strategy. Preclinical studies in both the KPC mouse model as well as patient-derived xenografts have shown blocking the MAPK pathway at MEK results in a decrease of cell proliferation and a subsequent halt in tumor growth (Collisson et al., 2012; Walters et al., 2013). Additionally, several MEK inhibitors are currently in clinical trials for solid tumors (http://www.clinicaltrials.gov/).

Similarly, inhibitors of both PI3K and AKT have been developed (Engelman, 2009). While inhibition of PI3K is complicated by the fact that there are multiple isoforms of the protein (for review see Vanhaesebroeck et al., 2010), and not all isoforms interact with Ras (Fritsch et al., 2013), preliminary studies in KPC mice show reduced proliferation and tumor growth upon PI3K inhibition (Eser et al., 2013). However, these results are not consistent across all preclinical models. Tumors in a xenograft model of pancreatic cancer were more sensitive to the blockade of MEK than PI3K (Hofmann et al., 2012), but treatment of xenografts with MEK and AKT inhibitors in combination increased the sensitivity of the tumors to radiation (Williams et al., 2012). The potential of MAPK and PI3K inhibition, alone or in combination, will likely be explored further in pancreatic cancer in the near future.

In addition to the MAPK and PI3K pathways, other Kras effectors have been shown to be active and functionally linked to pancreatic carcinogenesis (for review see Vigil et al., 2010). Inhibitors for components of those pathways, such as RalGDS, have been described (Gus-Brautbar et al., 2012). It will be one of the upcoming challenges to determine the relative importance of the different Kras effectors at different stages of the disease, and in individual cases of pancreatic cancer.

## Feedback mechanisms that regulate Kras activity

Since pancreatic cancer is associated with a mutant, constitutively active form of Kras, it has been supposed that Ras activity is constantly high in tumor cells. However, studies in mouse models have led to the surprising observation that, even when mutant Kras is present in every single cell of the pancreas from the beginning of the organ's embryonic development, the activity of downstream effectors of Kras is not elevated compared to the control pancreas. In fact, elevated activity of Kras effectors is first observed when the initial morphological alterations occur. Several recent studies have pointed at the need for positive feedback mechanisms to induce and maintain high Kras activity. For instance, ligand-driven EGFR activation is sufficient to activate Kras signaling, and is required for pancreatic carcinogenesis at least during the initial stages (Ardito et al., 2012; Navas et al., 2012). Other mechanisms of Kras activation include inflammatory stimuli such as those provided by the Nfκ B pathway (Daniluk et al., 2012), as well as IL6 (Zhang et al., 2013a). Finally, signaling pathways such as Wnt cross-talk with Kras to activate the MAPK cascade (Zhang et al., 2013b). The relative importance of these signaling pathways and their requirement at later stages of carcinogenesis need to be studied in further detail. If needed in invasive tumors, upstream regulators of Kras might provide additional therapeutic strategies, to complement direct targeting of Kras and inhibition of downstream effectors.

## Conclusions and open questions

The association of mutant Kras and pancreatic cancer has been known for decades, and validated by recent genome-wide sequencing studies. Our understanding of the regulation of Kras activity in pancreatic cancer has increased recently thanks to studies made possible by mouse models that mimic the human disease. Moreover, a series of discoveries has brought to light the multiple roles of Kras in pancreatic cancer, ranging from cell metabolism to interaction with the tumor stroma. Kras is a key oncogene during the onset of pancreatic cancer, and it is still required—at least in a subset of tumors—in invasive mouse and human pancreatic cancer. While resistance to Kras inhibition has been observed experimentally, it is still likely that direct inhibition of Kras would have at least a de-bulking effect on pancreatic tumors. This approach might be tested in the clinic in the near future, since new small molecule inhibitors for Kras are emerging. Mouse models provide a cautionary tale, as they indicate that resistance to Kras inhibition might eventually arise. Thus, it will be important to understand the mechanisms of acquired resistance and devise ways to eradicate pancreatic cancer.

A different set of open questions relates to personalized medicine for pancreatic cancer. Recent studies have indicated that human pancreatic tumors might be subdivided in different subsets with different biological characteristics and different susceptibility to Kras inhibition (Collisson et al., 2011). Moreover, pancreatic cancer cell lines have been shown to have different expression of cellular kinases, and have unique susceptibility to inhibition of those kinases (Kothari et al., 2013). The appropriate targeting of individual tumors might therefore depend on their individual characteristics.

Finally, successful translation of basic research findings to the clinic necessarily relies on identifying appropriate pre-clinical models. Traditional subcutaneous transplantation of human cell lines in immunocompromised mice has revealed poor predictive value. A significant advancement over traditional, cell line based transplantation models, is the use of patient-derived primary samples that represent individual tumors. When orthotopically transplanted in the pancreas, patient-derived xenografts maintain, at least in part, the microenvironment of the original tumor, with the caveat of lacking an intact immune system. Genetically engineered mice recapitulate the step-wise progression of the human disease and have an intact immune system and tumor microenvironment. Their clinical predictive value will however have to be carefully evaluated with controlled studies, and further optimization might be necessary to improve their translational potential. Going forward, a combination of primary-tumor based xenografts and genetically engineered mice might need to be used for preclinical validation of any new thearapeutic.

### Conflict of interest statement

The authors declare that the research was conducted in the absence of any commercial or financial relationships that could be construed as a potential conflict of interest.
